# Sea turtle (Reptilia, Testudines) diversity and occurrence in the Azores Archipelago (NE Atlantic)

**DOI:** 10.3897/BDJ.11.e98589

**Published:** 2023-02-16

**Authors:** Luís M. D. Barcelos, Frederic Vandeperre, Hugo Parra, João Pedro Barreiros

**Affiliations:** 1 cE3c- Centre for Ecology, Evolution and Environmental Changes, Azorean Biodiversity Group, CHANGE – Global Change and Sustainability Institute, School of Agrarian and Environmental Sciences, University of the Azores, Rua Capitão João d ´Ávila, Pico da Urze, Angra do Heroísmo, 9700-042, Terceira, Azores, Portugal, Portugal cE3c- Centre for Ecology, Evolution and Environmental Changes, Azorean Biodiversity Group, CHANGE – Global Change and Sustainability Institute School of Agrarian and Environmental Sciences, University of the Azores, Rua Capitão João d ´Ávila, Pico da Urze, Angra do Heroísmo, 9700-042, Terceira, Azores, Portugal Portugal; 2 Department of Oceanography and Fisheries, University of the Azores, Horta, Portugal Department of Oceanography and Fisheries, University of the Azores Horta Portugal; 3 IUCN - International Union for the Conservation of Nature, Groupers and Wrasses Specialist Group, Hong Kong, China IUCN - International Union for the Conservation of Nature, Groupers and Wrasses Specialist Group Hong Kong China

**Keywords:** marine vertebrates, marine turtles, Atlantic, sightings, occurrences

## Abstract

**Background:**

Six species of marine turtles occur in the Azores Archipelago. The loggerhead, *Carettacaretta* (Linnaeus, 1758), is by far the most common species and is being constantly monitored and tagged by a joint project between the University of the Azores and the University of Florida since 1989. With the implementation of the tuna fishery observers (for dolphin safe seals), an increment of sea turtle reports has been verified as expected. The leather back turtle, *Dermochelyscoriacea* (Vandelli, 1761) is the second most observed species in the Azores' EEZ, a fact probably also linked to the tuna fishery observation programme. All other species are occasional/vagrant albeit the green turtle, *Cheloniamydas* (Linnaeus, 1758) is more commonly seen than the others. Historically, sea turtles were occasionally taken for food in specific fishing villages and ports. Since 1986, sea turtles, as well as all marine mammals, are fully protected in the Azores although human-related activities (e.g. plastics, discarded fishing gear) do generate serious injuries and deaths.

**New information:**

In this paper, we update sea turtle species' checklist for the Azores and give detailed geographic coordinates on their known occurrences.

## Introduction

**Figure 1.**
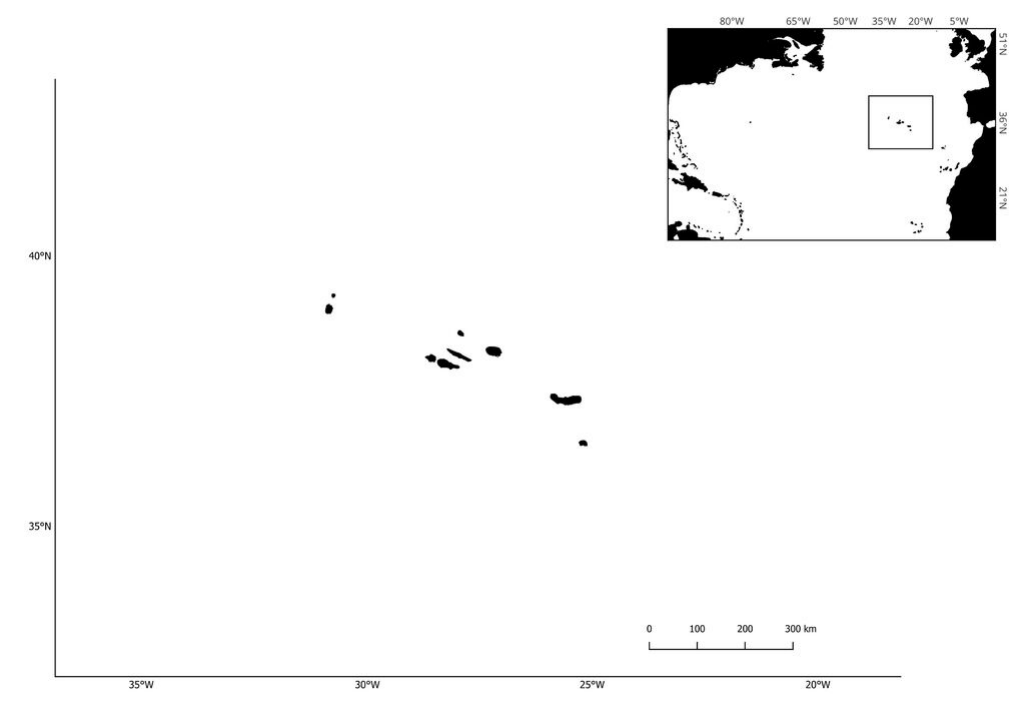
Azores Archipelago location in the North Atlantic.

The Azores form a remote, volcanic archipelago located in the Northeast Atlantic ocean, roughly halfway between Europe and North America (Fig. 1). This location makes the surrounding waters a biodiversity oasis namely for marine vertebrates. Although sea turtles are fully protected in the Azores, human activities (e.g. plastics and discarded/lost fishing gear, collision with ships) do cause serious injuries and deaths (see Barreiros and Barcelos (2001), Barreiros and Raykov (2014)). The recent confirmed report of the Olive Ridley, *Lepidochelysolivacea* (Eschscholtz, 1829) (Barcelos et al. 2021), increases the number of sea turtle species' in the Azores to six (Barcelos et al. 2022), which means all Atlantic species. This was the first report of a new species within the archipelago's EEZ (Exclusive Economic Zone) since 1990, when Bolton and Martins (1990), reported the presence of *Lepidochelyskempii* (Garman, 1880). The seventh known species, *Natatordepressus* (Garman, 1880), has a restricted distribution centred in northern Australia and southern Papua New Guinea.

Globally and according to the IUCN Red List, turtle populations are threatened, their statuses ranging from Vulnerable to Critically Endangered (see Table 1). Hence, it is increasingly important to have information on their distribution and occurrences, in order to carry out correct and precise habitat management.

## General description

### Purpose

**Figure 2.**
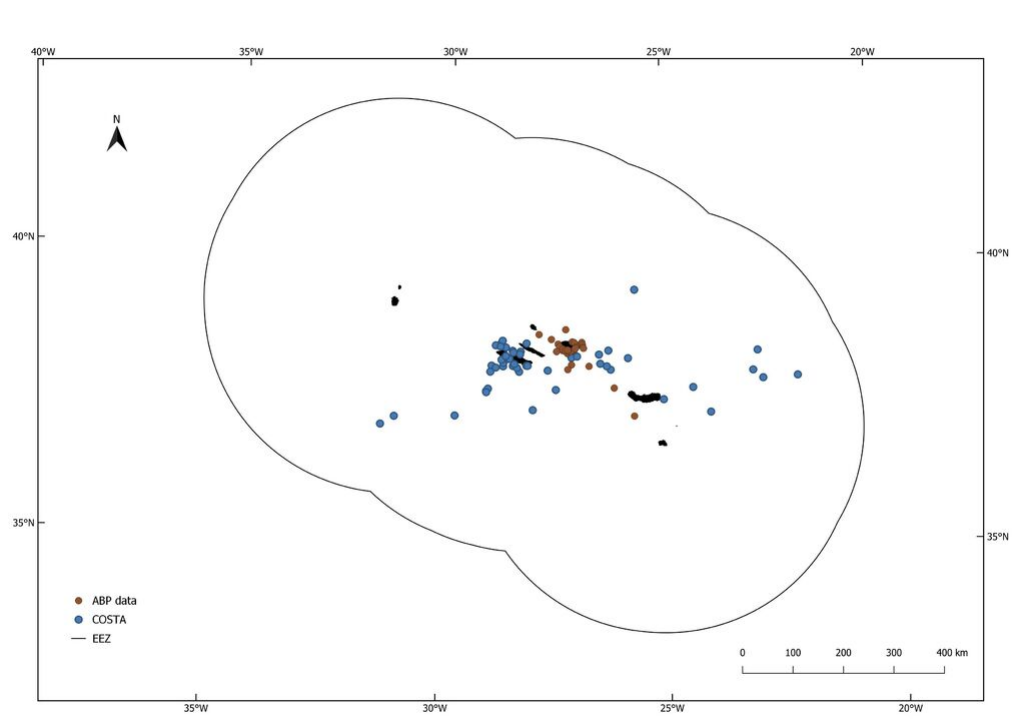
Sea turtle sightings in Azores EEZ. Blue - COSTA database; Red - Azores Bioportal database.

To consolidate the list of species previously published in GBIF (Barcelos et al. 2022) presenting information on methodology and occurrence records. A general overview of the data used (COSTA, GBA-JPB and GBA-LB) can be seen in Fig. 2. All the database and additional information can be found in Barcelos and Barreiros (2022).

### Additional information

The information contained in Fig. 2 and in Suppl. material 1 refers to only four of the six species that occur in the Azores. This is due to the fact that both *Lepidochelyskempii* (Garman, 1880) and *Eretmochelysimbricata* (Linnaeus, 1766), were not found by any of the authors during the data collection.

## Project description

### Title

AZORESBIOPORTAL - PORBIOTA

### Study area description

Azores EEZ

### Design description

The Azorean Biodiversity Portal E-Infrastructure (https://azoresbioportal.uac.pt/) was approved by FCT for the National Research Infrastructure in the Roadmap. The approval of Azorean Biodiversity Portal by the Portuguese E-Infrastructure Roadmap, guaranteed financial support between 2019 and 2021 and the improvement of the Portal and new products. This is quite an important achievement for this regional Biodiversity Portal. The Azorean Biodiversity Portal (ABP) is a key e-infrastructure for the integrated management of biodiversity data of the Azores, providing a large number of specialised services supporting research, policy and education (Borges et al. 2010). The evaluators considered that the submitted proposal lists some significant policy integration opportunities with Azorean government using the portal as part of its conservation activities for protected areas, as well as for the sustainable management of biodiversity relating to agriculture, forestry and fisheries. This was the first Biodiversity Portal in Portugal, starting in 2008 and the only one which provides easy access to island biodiversity data (Borges et al. 2010). ABP is currently recognised as a valuable outreach, management and conservation tool for all who work in science and protection of biodiversity. The large number of visits per day, the numerous international scientific collaborations, resulting in publications and academic theses and the connection with other prestigious databases demonstrate the Portal’s scientific quality as well as its general appeal. This project was initiated in 2008 under the leadership of researchers from the Azorean Biodiversity Group (CITA_A; currently cE3c -Azorean Biodiversity Group), based in the formerly Dept. of Agrarian Sciences (currently School of Agrarian & Environmental Sciences) in Terceira Island (Azores) and included also the collaboration with researchers from the CIBIO-Azores, based in the formerly Dept. of Biology of the Univ. of Azores (currently School of Sciences & Technology) and more recently researchers from OKEANUS-DOP in Horta. At this moment, the Portal is being funded by the Azorean Science Ministry (Azores PO 2020 - ACORES-01-0145-FEDER-000072). The main ABP action lines are to: improve the informatics system of the e-infrastructure to allow complex queries and improve user-friendliness; guarantee a rigorous classification for every species, providing updated comprehensive checklists, ensuring accuracy on the compilation of biogeographical information - this is the backbone of the Portal and all its products and services; provide innovative biodiversity analytical tools for both researchers and community members and invite them to contribute data to the Portal, establishing effective science communication.

### Funding

Funding Institutions: AZORESBIOPORTAL – PORBIOTA (Azores PO 2020 - ACORES-01-0145-FEDER-000072) TOTAL BUDGET: 299,901.83€ EU Support: 254,916.56€. This project was financed by FEDER in 85% and by Azorean Public funds by 15% through Operational Program Azores 2020. This work is also funded by FEDER funds through the COMPETE 2020 Programme and National Funds through FCT - Portuguese Foundation for Science and Technology under the Research Infrastructure PORBIOTA - Portuguese E-Infrastructure for Information and Research on Biodiversity, project number POCI-01-0145-FEDER-022127.

For the period 2022-2023- Portal da Biodiversidade dos Açores (2022-2023) - PO Azores Project - M1.1.A/INFRAEST CIENT/001/2022.

Open access will be supported by the project FCT-UIDB/00329/2020-2024 (Thematic Line 1 – integrated ecological assessment of environmental change on biodiversity).

## Sampling methods

### Sampling description

Bycatch data from 2008 to 2010 were recorded by fisheries observers onboard Portuguese commercial longline vessels, under the framework of the EU FP7 project MADE, Mitigating adverse ecological impacts of open ocean fisheries.

Bycatch data from 2016 to 2018 were recorded by fisheries observers onboard Portuguese commercial longline vessels, under the framework of COSTA project - Consolidating Sea Turtle Conservation in the Azores.

Sea turtle data from 2017 to 2020 were recorded by Azorean whale watching companies and researchers from IMAR-Institute of Marine Research under the scope of the sea turtle tagging programme coordinated by the Cooperative Marine Turtle Tagging Program (CMTTP) and maintained by the Archie Carr Center for Sea Turtle Research (ACCSTR).

GBA-LB data were collected between 2007 and 2019, by the first author, during whale watching tours.

GBA-JPB data were collected during systematic tag/release of marine turtles since 1990.

### Quality control

All persons involved on the handling of sea turtles are under permits issued by the Azores regional government.

## Geographic coverage

### Description

Azores' Exclusive Economic Zone (EEZ)

### Coordinates

33.6536 and 43.15985 Latitude; -35.493583 and -20.458427 Longitude.

## Taxonomic coverage

### Description

Sea Turtles

### Taxa included

**Table:** 

Rank	Scientific Name	Common Name
kingdom	Animalia	Animals
phylum	Chordata	
class	Reptilia	Reptiles
order	Testudines	Turtles
suborder	Cryptodira	
family	Dermochelyidae	
family	Cheloniidae	
genus	*Dermochelys*	
genus	*Lepidochelys*	
genus	*Eretmochelys*	
genus	*Chelonia*	
genus	*Caretta*	

## Usage licence

### Usage licence

Creative Commons Public Domain Waiver (CC-Zero)

## Data resources

### Data package title

Azores Sea turtles updated checklist

### Resource link

http://ipt.gbif.pt/ipt/resource?r=azores_seaturtles_checklist&v=1.2

### Alternative identifiers

http://ipt.gbif.pt/ipt/resource?r=azores_seaturtles_checklist

### Number of data sets

2

### Data set 1.

#### Data set name

Azores Sea turtles updated checklist

#### Data format

DarwinCore

#### Character set

UTF-8

#### Download URL

http://ipt.gbif.pt/ipt/resource?r=azores_seaturtles_checklist&v=1.2

#### Data format version

1.2

#### Description

Checklist of the sea turtles recorded in the Azores Archipelago.

**Data set 1.** 

Column label	Column description
ID	The Identifier for the line in the dataset.
taxonID	A global unique identifier for the taxon (name in a classification).
parentNameUsageID	An identifier for the name usage of the direct, most proximate higher-rank parent taxon of the scientificName.
scientificName	The full scientific name, with authorship and date information.
parentNameUsage	The name of the direct, most proximate higher-rank parent taxon.
kingdom	kingdom.
phylum	phylum.
class	class.
order	order.
family	family.
genus	genus.
specificEpithet	The name of the first or species epithet of the scientific Name.
taxonRank	The taxonomic rank of the most specific name in the scientific Name.
scientificNameAuthorship	The authorship information for the scientificName formatted according to the conventions of the applicable nomenclatural Code.
references	A related resource that is referenced, cited or otherwise pointed to by the described resource.

### Data set 2.

#### Data set name

Occurrence of sea turtles in the Azores Archipelago

#### Data format

DarwinCore

#### Character set

UTF-8

#### Download URL

http://ipt.gbif.pt/ipt/resource?r=occurence_seaturtles_azores&v=1.5

#### Data format version

1.5

#### Description

Information on distribution and occurrence of species, originating from sightings from fishing vessels, tourist activities and occurrences in coastal areas as well as fishing bycatch. These data can be consulted at Suppl. material 1

**Data set 2.** 

Column label	Column description
occurrenceID	An identifier for the Occurrence that makes the occurrenceID globally unique.
basisOfRecord	The specific nature of the data record.
eventDate	The date or interval during which an Event occurred. For occurrences, this is the date when the event was recorded.
scientificName	The full scientific name, with authorship and date information.
kingdom	kingdom.
phylum	phylum.
class	class.
order	order.
family	family.
genus	genus.
specificEpithet	The name of the first or species epithet of the scientificName.
scientificNameAuthorship	The authorship information for the scientificName formatted according to the conventions of the applicable nomenclaturalCode.
taxonRank	The taxonomic rank of the most specific name in the scientificName.
decimalLongitude	The geographic longitude (in decimal degrees, using the spatial reference system given in geodeticDatum) of the geographic centre of a Location. Positive values are east of the Greenwich Meridian, negative values are west of it. Legal values lie between -180 and 180, inclusive.
decimalLatitude	The geographic latitude (in decimal degrees, using the spatial reference system given in geodeticDatum) of the geographic centre of a Location. Positive values are north of the Equator, negative values are south of it. Legal values lie between -90 and 90, inclusive.
geodeticDatum	The ellipsoid, geodetic datum or spatial reference system (SRS) upon which the geographic coordinates given in decimalLatitude and decimalLongitude are based.
coordinateUncertaintyInMetres	The horizontal distance (in metres) from the given decimalLatitude and decimalLongitude describing the smallest circle containing the whole of the Location.
country	The name of the country or major administrative unit in which the Location occurs.
countryCode	The standard code for the country in which the Location occurs.
islandGroup	The name of the island group in which the Location occurs.
establishmentMeans	The process by which the biological individual(s) represented in the Occurrence became established at the location.

## Supplementary Material

6712519E-CA65-5034-9147-565B09D625A4 10.3897/BDJ.11.e98589.suppl1 Supplementary material 1 Occurrences and taxonomic information Data type occurrences Brief description Data on the occurrence of sea turtles in the RACA, COSTA and Azorean Biodiversity Group databases. These data are accessible in GBIF through: http://ipt.gbif.pt/ipt/resource?r=occurence_seaturtles_azores File: oo_779505.csv https://binary.pensoft.net/file/779505 Luís MD Barcelos, João P Barreiros

## Figures and Tables

**Table 1.** IUCN Red List classification of the six species present in Azores EEZ.

**Scientific Name**	**IUCN classification**	**IUCN classification Source**
*Carettacaretta* (Linnaeus, 1758)	vulnerable	Casale and Tucker (2017)
*Cheloniamydas* (Linnaeus, 1758)	endangered	Seminoff (2004)
*Lepidochelysolivacea* (Eschscholtz, 1829)	vulnerable	Abreu-Grobois and Plotkin (2008)
*Dermochelyscoriacea* (Vandelli, 1761)	vulnerable	Wallace et al. (2013)
*Lepidochelyskempii* (Garman, 1880)	critically endangered	Wibbels and Bevan (2019)
*Eretmochelysimbricata* (Linnaeus, 1766)	critically endangered	Mortimer and Donnelly (2008)
